# Embryology of the Fascial System

**DOI:** 10.7759/cureus.10134

**Published:** 2020-08-30

**Authors:** Leonardo Vieira

**Affiliations:** 1 Osteopathy, Brazilian Academy of Fascias, Belo Horizonte, BRA

**Keywords:** embryology, fascial system, fascia, human development, neuro-fasciagenic

## Abstract

The fascial system is a link between the various body systems. Understanding the embryonic formation of the fascial system contributes to understanding the development of the whole body, helping to understand clinical phenomena. The text presents the concept of the fascial system and its interactions with the neural system. We describe the formation of musculoskeletal fascia from somites and mesenchymal cells of the cranial neural crest. Differences in the formation of the head, neck, trunk, and limbs and their respective embryonic relationships are presented.

We detail the formation of visceral fascia and their corresponding innervations, from the tongue to the final portion of the digestive tract; the development of the genitourinary system that occurs later in the celomic cavity; and the formation of the cardiocirculatory and respiratory systems, with the development of their respective envelopes, associated with the corresponding innervation. The text covers the embryology of neural fasciae, both at the level of the central and peripheral nervous system. Finally, the development of derme and pannicular fascia is presented.

## Introduction and background

The definition of the term fascia is not yet a consensus among the various research groups on the subject. Following the definition of the Fascia Research Society, published in 2014, fascia consists of the entire sheath of anatomically dissectible connective tissue. In addition, the same group presented a broader definition of a large fascial system: "The Fascial System consists of a three-dimensional continuum of soft connective tissues, containing fibrous, loose and dense collagen that permeates the body. It incorporates elements such as adipose tissue, adventitia, neurovascular sheaths, aponeurosis, deep and superficial fasciae, epineurium, joint capsules, ligaments, membranes, meninges, myofascial expansions, periosteum, retinaculum, septum, tendon, visceral fascia and intermuscular connective tissue, including endomysium/epimysium/perimysium. Fascial system involves, intertwines and interpenetrates all organs, muscles, bones and nerve fibers, providing the body with a functional structure and providing an environment that allows all systems in the body to operate in an integrated manner” [1]. As the body’s liquid system is formed from mesodermal mesenchymal development and influences the biotensegrity of the entire body, some authors propose the integration of blood and plasma as part of this great fascial system, receiving the designation of liquid fascia [2].

In 2015, Paolo Tozzi presented the Neurofasciogenic Model to the world, which highlights the relationship between the fascial and neural system with the functioning of all the systems in the body. He considers this neurofascial interaction as key in the complex interactions between the various tissues and organs of the body [3].

Understanding the origin of this entire neurofascial system is fundamental to expand the possibilities to evaluate and intervene more efficiently in such a complex system. Understanding the embryology and the formation relationships of these tissues expands the understanding of their relationship with clinical changes, interconnecting the superficial, deep, visceral, neural, and vascular fascias.

Embryology is described as the area of ​​biological science that studies the formation of an animal’s organs and systems from a cell [4]. It starts after fertilization until the eighth week of formation. The anatomy presents several variations between individuals of the same species. Embryology, under normal conditions, presents a similar pattern of formation of individuals, which even respects the order of phylogenetic evolution of the species [5]. We have two main lines of observation of embryology, one genetic and the other metabolic. With advances in technology, genetic studies have gained great visibility in the research scenario. However, works such as the ones presented by Donald Ingber, a Harvard biologist, demonstrate that the genetic code responds to the stimulus and the relationship of the cell with the extracellular environment. This concept is presented as epigenetics and establishes a relationship between genetics and the influences of the external environment, unifying the two previous perspectives [6]. The purpose of this paper is to make a review regarding the embryology formation of the human species in the formation of the fascial system and its interconnection with all systems, prioritizing metabolic relationships without, at any time, diminishing the genetic importance in the individual’s formation process.

Works by Streeter in 1942 [7] and O’Rahilly and Müller in 1987 [8] standardized a system of chronological division of evolution in 23 different stages. These stages were delimited through the development of the structures and are called Carnegie Stages, a reference to the Carnegie Institute, an organization created to support scientific investments in the area, based in Washington D.C., USA.

## Review

Somitogenesis

**Figure 1:**
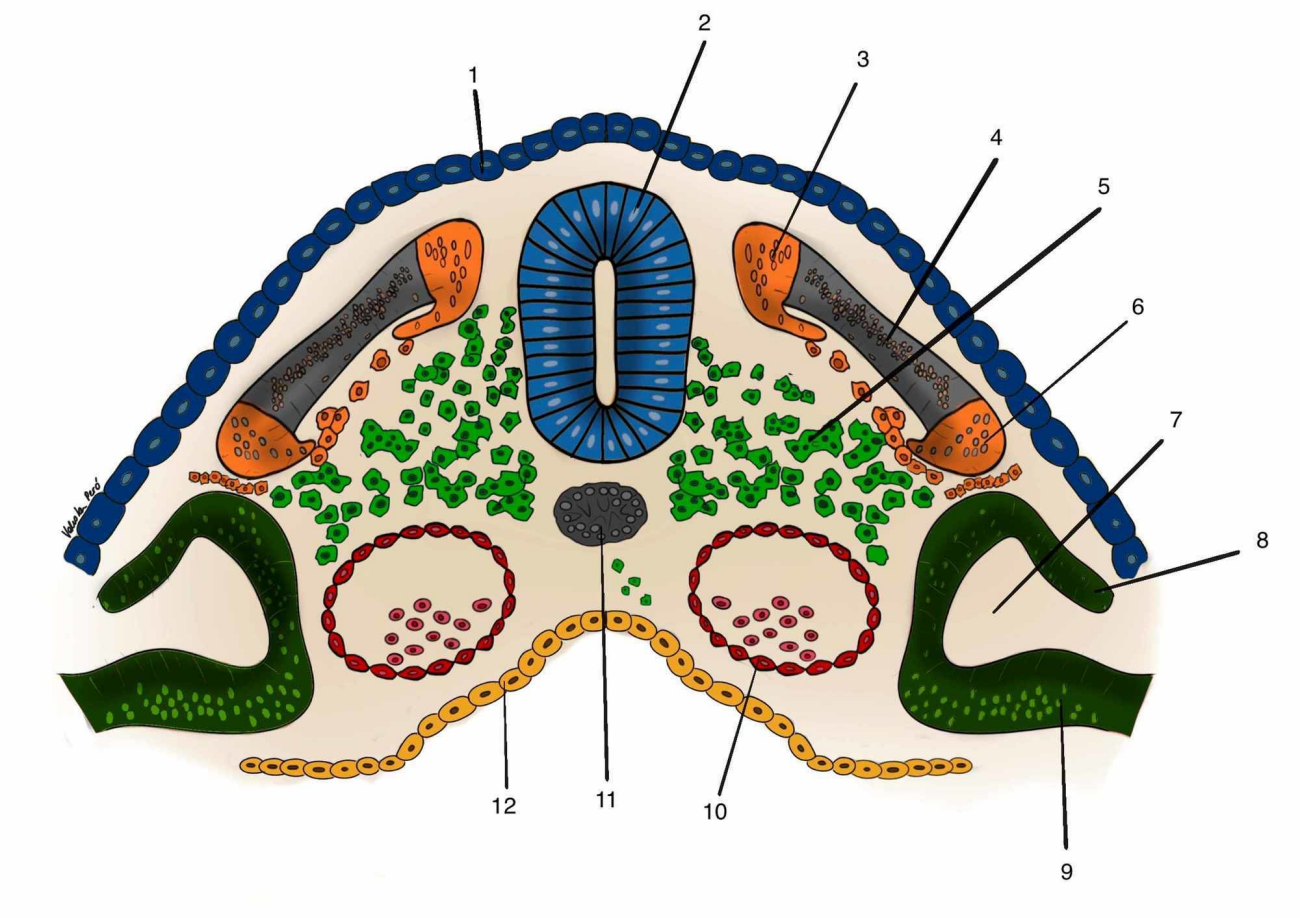
A somite and its components 1: layer of cells from ectoderm that will develop epidermis; 2: neural tube; 3: epimere, part of myotome; 4: dermatome; 5: sclerotome; 6: hypomere, part of myotome; 7: celoma cavity; 8: somatopleure; 9: splancnopleure; 10: abdominal aorta; 11: notocord. Author's personal database image

On the twentieth day of embryonic life, the embryo begins a segmentation process called somitogenesis. After the process has started, every 90 minutes on average, a new somite is formed until a total of 44 pairs are formed [9]. According to Blechschmidt and Gasser [10], this segmentation occurs from the formation of transverse arteries, originating from the primitive dorsal aortic arteries and cardinal veins. At the same time that they nourish the high speed of cell growth of the cells of the neural tube, they also form a restrictive apparatus that limits this speed by forming the segmentation process of the entire neural tube with the exception of the cranial part. Somites are formed from the paraxial mesoderm and is characterized by having three basic structures: sclerotome, dermatome and myotome [9] (Figure 1).

The sclerotome will give rise to the bones of the body, with the exception of the skull bones and hyoid bone, which develop from the cells of the cranial neural crest [11].

Dermatomes, together with somatopleural cells, will give rise to the dermis, superficial fascia, and layers of subcutaneous fat. The dermatome is innervated from the anterior and posterior branches of the spinal nerve. The skin and superficial fascia of the posterior region of the body will be innervated by the posterior branch of the spinal nerve. The skin and superficial fascia of the anterior part of the body carries the innervation of the anterior branch of the spinal nerve.

Myotomes develop the muscles and deep fascia of the body, with the exception of the fascia of the face and the anterior neck that develop from the mesenchymal cells of the cranial neural crest, originating from the ectoderm [11].

Muscles and fasciae of movement

The movement fascia basically divide into two structures: the epimysial fascia and the deep fascia. The epimysial fascia is the fascia that surrounds and organizes a single muscle. It is represented by the epimysium that surrounds the muscle externally, the perimysium that organizes muscle fascicles, and the endomysium that surrounds a muscle fiber. Anatomically, the epimysial fasciae are not dissectible structures. We also have the deep fascia that involves muscle groups organizing the synchronism of the movement at a distance. It has the ability to transmit strength and save energy [12].

All components of the movement system have a common embryonic origin, including capsules, ligaments, tendons, aponeuroses, retinacula. This all constitutes a unit called the fascial system of the movement. With the exception of the face and neck, all connective tissue, muscles and bones and movement fasciae derive from the paraxial mesoderm, more precisely from the myotome, which is one of the structures that make up the somite [11].

**Figure 2:**
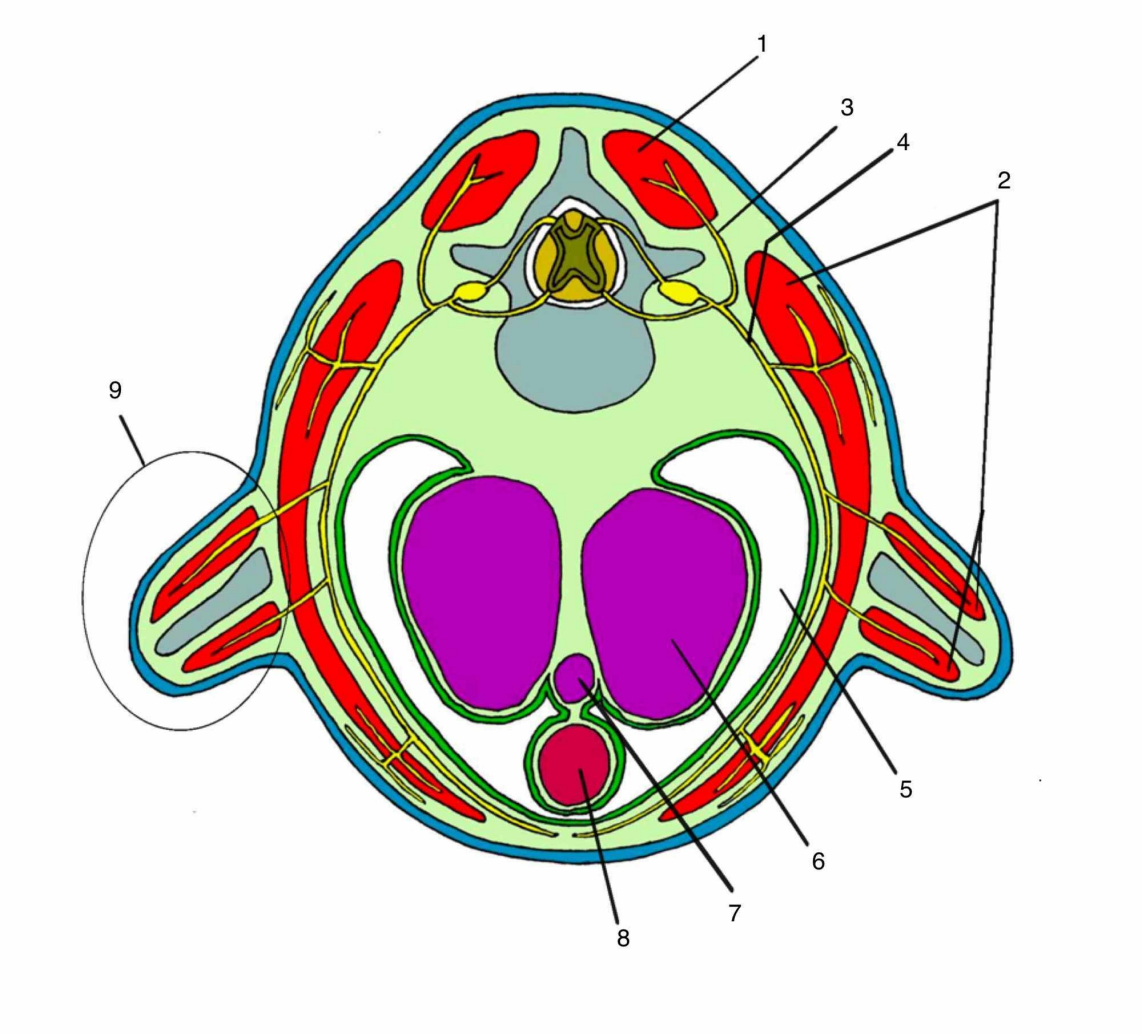
Transversal section of right thoracic spine 1: epimere; 2: hypomere; 3: posterior ramus of spinal nerve; 4: anterior ramus of spinal nerve; 5: celoma cavity; 6: lung; 7: trachea; 8: esophagus; 9: primary formation of limb Author's personal database image

Myotomes subdivide into two parts: the epimer and the hypomer. The epimer is formed by the mesodermal cells that are located in the dorsal region of the embryo and that develop from the metabolic relationships of the growth of the neural tube [10]. From the epimer the fasciae and muscles of the trunk are formed and will be located in the future after the transverse vertebral process. They will mainly form the extensor muscles of the trunk and head and their respective fascial sheaths. The erector muscles of the spine, the intertransversals, the interspinals, and their respective fasciae develop from the epimer [13] (Figure 2).

The hypomer is the part of the myotome that forms in the most ventral region of the somite. It consists of an organization of mesodermal cells that develop according to the metabolic relationship of the reduction of the celomic cavity from the development of the digestive and respiratory tract. It will give rise to the muscles and fasciae of movement of the anterior region, to the transverse vertebral processes and limbs [13]. The three layers of the deep fascia of the trunk, with their respective muscles, are derived from the hypomer, with the exception of the trapezius and sternocleidomastoid muscles.

Around the 24th day of life, the thickening of ectodermal and mesodermal cells is evident in the region corresponding to the eight, nine, and 10 somites of the embryo. This will result in the development of buds that will originate scapular girdle and upper limbs, in Carnegie’s stage 12. About two days later, the same phenomenon is repeated at the caudal level of the embryo, initiating the formation of the pair of buds that originated the lower limbs and pelvic girdle, already in Carnegie’s stage 13. The muscles and fascia of movement of the limbs will develop exclusively from the hypomer. So the extending compartments are formed from a spiraling process of the hypomer [10] (Figure 2).

Formation of the limbs

The formation of buds is a process subsequent to the process of visceral development. The repositioning of the transverse septum with the development of the embryo is a key event in several metabolic changes that occur in the future thoracic region. The heart and liver tend to present an anterior displacement, freeing space in its posterior part, occupied by the coelomic cavity. As the organs in the thoracic region grow, especially the lungs, there is a considerable decrease in the coelomic cavity in the thoracic region [14]. With this, several chemical substances are released in the extracellular matrix of the region’s mesoderm. These substances alter the relationship of ectodermal and mesodermal cells in the region of the future “skin” of the embryo (respectively, epidermis and dermis), generating a cell thickening in the respective region, called the ectodermal ring [15]. These relationships form the bud pair that will develop in the upper limbs. The hypomer is located close to this region and is directly influenced by these relationships. It will be responsible for the formation of muscles and movement fascia of the limb region. As a result, the movement fasciae and muscles of this region are innervated exclusively by the anterior branches of the spinal nerves, which are part of the brachial plexus [16]. The repositioning of the transverse septum also promotes a reorganization in the retroperitoneal and pelvic structures. This event directly influences the development of the genitourinary system based on the organization of the intermediate mesoderm. The formation of the kidney, ureter and gonads occurs from the organization of metanephros, mesonephros, Wolff’s duct and Muller’s duct. This causes changes in the coelomic space of this region, causing the release of genetic factors that influence the formation of the pelvis and the pair of buds that will originate the lower limbs [14]. The myotome part of these buds is derived from the hypomer that carries the innervation of the anterior branch of the spinal nerve that originated the anterior part of the lumbosacral plexus [16].

Muscles and fasciae of the trunk, thoracolumbar fascia, rectus abdominis sheath

The deep fasciae, epimysial and trunk muscles develop from the axial mesoderm of the myotomes referring to the somitic formation [13]. The deep fasciae organize the trunk muscles into three continuous layers. The most superficial layer previously involves the pectoralis major and external oblique muscles. Subsequently it involves the latissimus dorsi, teres major and the trapezius muscles. The middle layer of the deep fascia involves the subclavian, pectoralis minor, serratus anterior, subscapularis, infraspinatus, supraspinatus, teres minor, rhomboids, serratus posterior superior, posterior-inferior and internal oblique muscles. The deep layer of the deep fascia surrounds the intercostal muscles, transverse to the sternum, and transverse to the abdomen [17]. All these muscles are formed from the hypomer, being innervated by anterior spinal nerves [13].

The sheath of the rectus abdominis forms a layer derived from the three layers of the deep fascia of the trunk, enveloping the rectus abdominis muscle. This structure, together with the thoracolumbar fascia, is responsible for controlling movement and multiplanar tension distribution across the trunk. It develops from the trunk receiving innervation from the anterior thoracic spinal nerves [18].

The thoracolumbar fascia is an important structure related to the transmission of force as well as synchronism between the pelvic and scapular girdle [19]. All three layers of the deep fascia of the trunk are found in the dorsal region and are reorganized involving the posterior muscle groups. It constitutes an extremely important region for the bipedal posture, being a fundamental part in the generation of stability and synchronism of the limbs with the trunk. It is a complex anatomy structure presenting several model proposals in the literature. From the perspective of the two-layer model, the transverse process of the lumbar vertebrae constitutes the point of reference for their division [13]. The anterior layer is in front of the lumbar transverses and involves the quadratus lumborum and psoas. This layer develops from the hypomer and has a great relationship with the diaphragm through the medial and lateral arcuate ligaments. It is related to the transversalis fascia and the renal fascia, synchronizing the trunk movement with the visceral movement [20]. It is innervated from the nerves of the lumbar plexus. The other layer is described after the transverse process and is subdivided into superficial and deep. The superficial is formed by the fascia of the great dorsal and contralateral gluteus maximus, being responsible for the synchronism between pelvic and scapular girdles. This layer develops through the hypomer receiving innervation from the anterior ramus of spinal nerves, the thoracodorsal nerve and the lower gluteal nerve belonging to the sacral branches [15]. The other part of the posterior layer of the thoracolumbar fascia is deeper, involving the spinal or paravertebral erectors formed by the spinal, longitudinal, iliocostalis, iliolumbar muscles, in addition to the intersegmental muscles such as the multifidus, intertransversal and interspinatus. They develop from the epimer, being innervated by the posterior branches of the spinal nerves presenting a segmental pattern [13].

Contrary to logic, the thoracolumbar fascia, being an important structure in movement, posture and stabilization, has little concentration of proprioceptors in its layers [21]. The most superficial posterior layer, formed by the fascia of the great dorsal and opposite gluteus maximus fascia, has the highest concentration of receptors, most of which are free nerve endings, that is, multimodal receptors. Under normal conditions, where the transmission of force occurs appropriately in the transverse and frontal plans in particular, these polymodal receptors assume a role as proprioceptors. A portion of individuals with chronic low back pain show a decrease in movement between these fascial layers [22]. The receptors, in this case, would function as nociceptors with potential for sensitization at the lumbosacral spinal levels through the afferences of the paravertebral and gluteal fasciae. Because of the innervation of the brachial plexus, C5-C6, of the great dorsal, the most superficial posterior layer of the thoracolumbar fascia would have the potential to sensitize the respective cervical levels, which may cause changes in movement and neck pain.

Alteration of myotomes in relation to dermatomes

The myotomes and dermatomes in the trunk coincide in the same vertebral segments, as they present innervation of the anterior and posterior branches of the spinal nerves [23]. When generating an overlay of the maps we have similar spinal regions related to the skin, muscles and movement fasciae. The dermis of the posterior region is innervated by the posterior branch of the spinal nerve. At a deeper level, in relation to the deep and muscular fasciae are derived from the epimer, a structure of the myotome innervated by the same posterior branch of the spinal nerve. In the lateral and anterior regions of the trunk, the skin receives innervation from the anterior branch of the spinal nerve. Similarly, it occurs with the muscles and fasciae of movement that develop through the hypomer [16].

However, the members are different. The dermis of the anterior arm and forearm is innervated by the anterior branches of the spinal nerve. The posterior region of the arm and forearm receives innervation from the posterior branch of the spinal nerve, following the pattern of organization of the trunk. The myotome is different. The muscles and fasciae of movement of the limbs are formed only from the hypomer by a process of spiraling [10]. In this case, the dorsal compartments are formed by the hypomer, presenting a pattern of innervation by the anterior spinal branches differing from the pattern of innervation of the dermatome.

As a result, the deep fascia and muscles will have different levels of spinal cord innervation in relation to the skin in the limb, pelvic and scapular girdles [23].

Deep fascia and muscles of the head and neck

**Figure 3:**
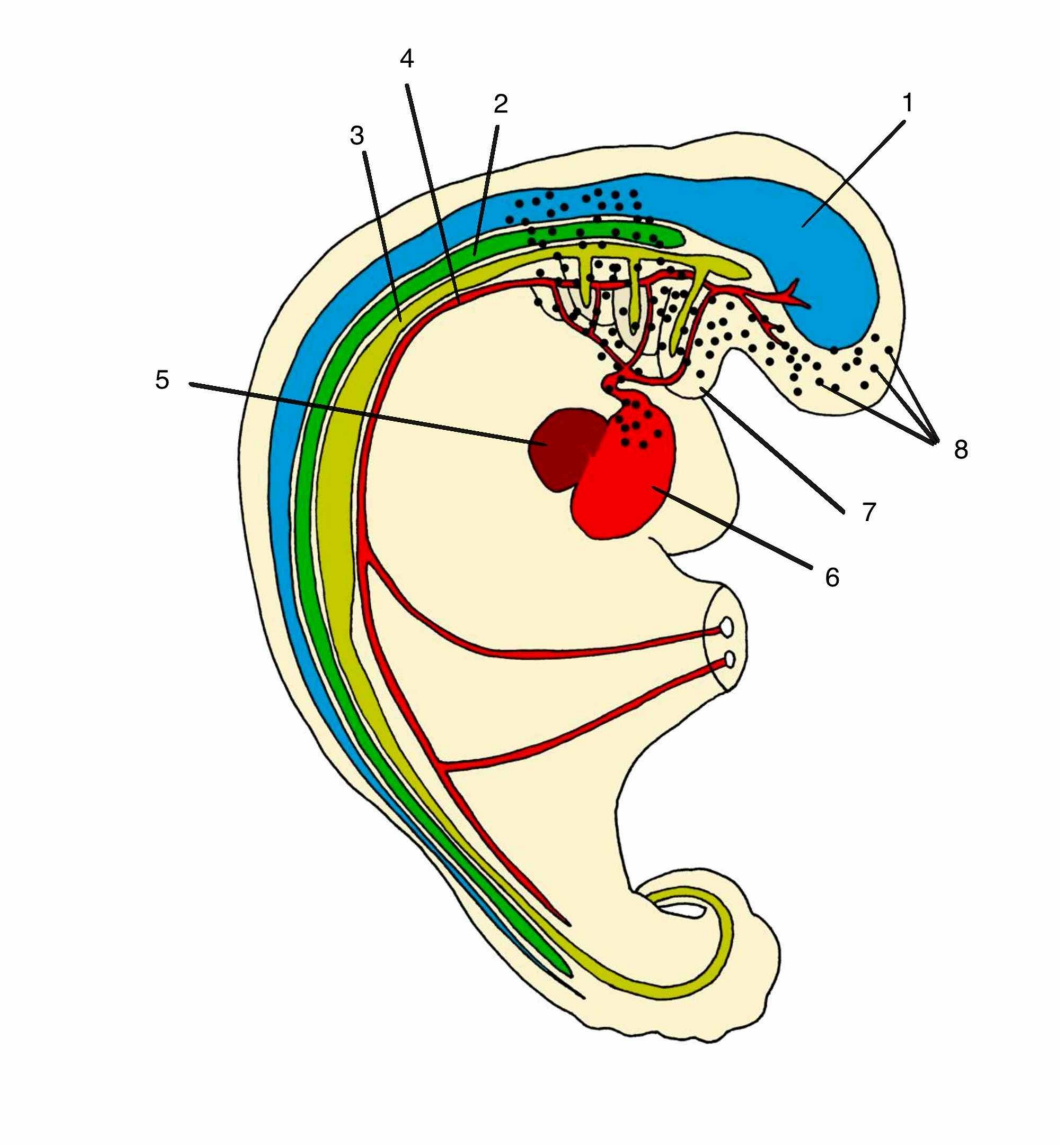
Cells of neural crest forming connective tissue of face and neck 1: Neural tube (ectoderm); 2: mesoderm tissue; 3: endoderm tissue; 4: primitive aortic; 5: liver; 6: heart; 7: pharyngeal arch; 8: cells of neural crest Author's personal database image

The connective tissue that will originate the bones, muscles and fascia of movement of the anterior region of the neck and face develops from the mesenchymal cells of the cranial neural crest [24] (Figure 3). These cells are attracted to the anterior region through the development of pharyngeal arches. The development of the endoderm of the primitive gut tube releases gene factors responsible for this cranial mesenchymal cell migration [25]. Then the origin of the connective tissue in this region will be formed by ectodermal and non-mesodermal cells as in the whole body. The rapid development of foregut (endoderm) generates a huge increase in metabolic demand in the cranial region. This generates an organization of the dorsal aortic artery forming the pharyngeal arteries. At the same time that the arteries nourish the tissues, they form an apparatus that restricts their growth because they do not keep pace with their development [10]. With this, pharyngeal arches are formed, segmentations of the cranial endodermal part of the digestive tract, accompanied by the adjacent mesoderm [26]. The first pharyngeal arch appears around the 20th day of life [9]. Its formation is influenced by the cephalization process with the first fold of the embryo. According to Blechschmidt and Gasser, this arch is influenced both by the development of the cranial ectoderm and by the development of the primitive gut tube [10]. At the bone level it forms the maxilla, the mandible, the zygomatic, part of the temporal bone and in the external auditory canal it forms the hammer and anvil. From it the muscles of mastication and the respective deep fasciae of the muscles are developed: deep temporal, masseter, lateral and medial pterygoid. The cranial pair V, trigeminal innervates the structures formed from the pharyngeal arch I [15].

The other pharyngeal arches are developed mainly due to the growth of the digestive tract. The second arch will form the mimic musculature and the fasciae of the face. In the face region, we cannot anatomically separate the superficial from the deep fascia. At the bone level, the body and lower process of the hyoid develop, and in the external ear, the stapes ossicle. The sinuses and tonsils develop from the folds and the space between the first and second arches. The structures originated by the second arch receive the innervation of the VII for cranial, facial nerve.

The third pharyngeal arch forms the upper pharyngeal constrictor muscle and part of the buccopharyngeal fascia. It receives glossopharyngeal innervation, pair IX [10]. The internal carotid artery develops from it. The sac of this arch will originate the thymus, and the lower part of the parathyroid.

From the fourth pharyngeal arch develop the cricoid, thyroid cartilage, the aortic arch and the right subclavian artery, laryngeal muscles and the upper part of the parathyroid gland. The soft palate, epiglottis, uvula, pharyngeal fasciae and the upper part of the digestive and respiratory tract, part of the sternum and ear canal. The structures are innervated by the vagus nerve, cranial pair X.

The fifth pharyngeal arch degenerates with the development of embryo [18]. The sixth pharyngeal arch is controversial in the literature. Some embryologists consider it part of the IV pair. It forms the middle and lower pharyngeal muscles and their respective fasciae. It also forms muscles of the larynx. The development of the trapezius and sternocleidomastoid (SCM) seems to be related to the development of this pharyngeal arch and occipital somites. Their structures are innervated by the accessory nerve, pair XI. The tongue is formed from the first four pharyngeal arches and its muscular part derives from the four occipital somites. Because it is such a complex structure, its embryonic formation will be described separately.

In the anterior part of the cervical, the fascia of movement and muscles are formed due to the great influence of the formation of the digestive tube and pharyngeal arches and consequently present continuity with the visceral fasciae, as is the case of the middle layer of the deep cervical fascia that surrounds the infrahyoid muscles and also the trachea, thyroid and parathyroid and are continuous forming the suspensory ligaments of the pleura and pericardium (Figure 3).

Tongue formation

The evolution of the tongue was important for the social evolutionary leap of humans [27]. In conjunction with other adaptations, it was fundamental for the articulation of different types of sounds. Its neurofascial connections interfere in the digestive, respiratory, phonation and movement, mainly in the upper cervical spine. It presents continuity with the movement of the floor of the mouth and cervical fasciae, base of the skull and dura mater and the fasciae of the digestive and respiratory tract [28].

The tongue is formed by joining structures derived from the first four pharyngeal arches. The anterior and lateral region of the tongue presents trigeminal innervation responsible for the perception of sensitivity. This same region has neural receptors responsible for capturing the flavor being innervated by the facial nerve. The tasting of the posterior part is carried out through receptors that carry their information through the glossopharyngeal nerve. The vagus nerve is responsible for the sensitive part of the posterior part of the tongue and epiglottis. It assists in the musculature of the tongue by innervating the palatoglossus. Of the four occipital somites, the musculature of the tongue is formed [15]. With the exception of the palatoglossus, all are innervated by the hypoglossus, cranial pair XII. In addition to motor function, it carries the afferent proprioceptive part of the tongue and the afferent part of the region of the dura mater anterior to the foramen magnum [29].

Heart: muscles and cardiac fasciae

The heart develops from the migration and differentiation of cells from six different origins in the human species. Until the beginning of the third week of formation, the main nutritional mechanism of the embryo occurs by diffusion between cells. From the third week onwards, a group of cells derived from epiblast progenitor cells are organized with the function of helping the distribution of nutrients in the embryo. These cells develop along the most cranial part of the primitive line [15]. Ectodermal mesenchymal cells together with mesodermal cells in the cranial region of the embryo undergo a differentiated process stimulated, mainly by the high metabolic rate of the cranial cells, both by the process of cerebralization, ectodermal and the process of development of foregut, an endoderm in the cranial region [11]. A primitive heart is formed out of this complex mechanism from the growing need for nutrition in the systems in formation [30]. The first heartbeats start around the 21st day. From the cranial fold of the embryo we have the formation of the cardiogenic region. In this region, the intra-embryonic cell is converted into a pericardial cavity. The cranial neural tube presents a very accelerated growth involving and bending over the pericardial cavity. Migration of mesenchymal cells from the cranial neural crest occurs to form a muscular part of the heart [31]. This phenomenon is called the first wave of mesenchymal growth. This phenomenon is likely to help in the formation of cardiac fasciae. This gives rise to the primary cardiogenic field that will undergo several transformations, over the four to eight weeks, promoted by the great speed of development of the embryo. After the formation of this cardiogenic field, these cells are induced by the pharyngeal endoderm underlying to modify, forming cardiac myoblasts and blood islets. These cells will initiate vasculogenesis by forming blood vessels and blood cells. At that moment, the cells form a horseshoe-shaped structure that will soon become a tube with endothelial organization covered by myoblasts. The primary cardiac body consists of the right and left horns of the venous sinuses, the primitive atrium, the atrioventricular canal, and the left primitive ventricle. Cardiac tube elongation, bending and curvature are moved by the second cardiac field. The right ventricle and the outflow tract become important acquisitions to the outflow. At the end of the influx, the second cardiac field contributes to the formation of the right and left atria and the atrial septums [9]. The heart develops from an organization in layers that form a continuous spiral. This generates great efficiency in the pumping function, saving a lot of energy [32]. In addition, the extracellular matrix of the cardiac connective tissue presents an array of collagen fibers orchestrated by fibroblasts, generating a synchronism of the entire cardiac tissue. With this arrangement, cardiomyocytes begin to optimize their function by spending less energy and increasing durability.

As the heart develops, the space of the cardiac cell is reduced, forming two layers of cardiac fascia, one more internal, called the visceral pericardium or cardiac investing fascia. The outermost represents the most superficial part of the coelom that forms the parietal pericardium or cardiac insertion fascia. From this layer and its relationship with the mesoderm of neighboring structures, ligaments that connect the heart with neighboring structures will be formed: sternopericardial ligaments, cardiophrenic ligaments. The repositioning of the transverse septum occurs in conjunction with the positioning of the heart in the mediastinum. These events are responsible for the formation of the suspensory ligaments of the pericardium formed from mesenchymal cells that constitute the middle cervical fascia [33]. At the beginning of the functioning of the primitive heart, there is no specific neural control for this new cluster of contractile cells to perform its function. Each cardiac cell functions as a pacemaker [9]. With the helical layout and the timing of the entire cardiac system results in great efficiency [32]. A sympathetic innervation network together with the vagus nerve develops orchestrating this complex set. This innervation has a local control through the sinoatrial node and the atrioventricular node and a control at the brainstem level, with specific neuronal nuclei [15].

Vascular and hematopoietic system

With embryonic development there is an increase in the flow of fluids resulting in an intense increase in metabolism. This causes a large amount of fluid currents. These currents preferentially occur in the extracellular matrix of the mesoderm, which is where the metabolic reactions take place in the embryo. Depending on the characteristics of this current, the mesenchymal cells are grouped and changing their shape and function forming the endothelial cells. These cells acquire a flattened shape due to the current pressure [10]. They are grouped by cellular junctions that allow selective passage of only a few substances forming the vessels. Thus, blood vessels appear in the first month of life [9]. Vasculogenesis, the process of forming new ones by organizing mesodermal cells, begins around the 17th day of life in the mesenchyma of the yolk sac. It starts at the splenic mesothermal and extends to the paraxial mesoderm. This fluidic relationship induces the formation of hemangioblasts that are progenitor cells of two specific lineages: hematopoietic cells and endothelial precursor cells. The first results from differentiation of mesenchymal cells of the yolk sac forming the first erythrocytes [9]. The liver is colonized by these cells in two waves, one on the 23rd and the other on the 30th day, becoming the main hematopoietic organ of the embryo and fetus. Near delivery, the bone marrow has developed enough to assume the role of this function. Endothelial precursor cells are initially generated in the yolk sac from hemangioblasts [9]. These cells group together to form blood islets, which are the designs of the first vessels. The splanchnic mesoderm together with the paraxial mesoderm will originate all the vessels in the body. The flow relationships with the mesodermal cells are preponderant for the differentiation of arteries and veins [10]. The main difference between them corresponds to the intracellular junctions, changing the permeability between the vessels. These junctions allow the arteries to hinder the entry of substances and the veins to hinder the exit. In addition, large caliber arteries have three muscle layers in their constitution while veins have only one. The veins have valves to prevent the return of blood, since they do not have a driving force like the arterial system [15].

The arterial system is formed from the dorsal aorta. The dorsal aorta are divided into posterolateral branches that will form the arteries of the entire skull, spine and aortic arch. The lateral branches will form the arteries to the adrenal, kidneys and gonads. The central branches will form the vitelline arteries and consequently the visceral, celiac, superior and inferior mesenteric arteries. And the umbilical artery will originate five lumbar pairs and part of the internal iliac artery [9]. The dorsal aortic arteries join in the thoracic region with the descent of the transverse septum and reorganization of the abdomen structures [10].

Cardinal veins will originate the entire cranial and medullary venous system, vertebral column, vena cava, azygos and hemiazygos. The umbilical veins will form the falciform and round ligaments, and the vitelline veins will originate the portal venous system [9].

All the derivation of the vascular layers comes from the mesoderm, both the endothelial, the muscular and the adventitial layer, which is considered the first fascial layer itself [33].

In 2014, Carla and Luigi Stecco proposed an organization for vascular fasciae in a similar way to the organization of visceral fasciae [33]. The innermost layer is called the investing fascia and the outermost is the insertion fascia. The vascular or adventitial investing fascia contains a greater proportion of elastin fibers and is related to the muscular layer of the vessels. The separation of the two vascular fascial layers is visible only in large vessels. In the cervical, the arterial insertion fascia is formed by the union of the three layers of the deep neck fascia, being called the carotid sheath [34]. In the upper part of the cervical, it is continuous with the fascia of the face and head, neurocranium [9]. In the thoracic it is continuous with the mediastinal vessels until the entrance of the aorta and joins, in the posterior part, the thoracic prevertebral fascia [33]. It crosses the transverse septum forming the aortic and vena cava gap. The aortic hiatus is continuous with the fascia of the diagfragmatic crura [35]. This directed flow is fundamental for morphogenesis throughout the body, since the shape of the internal structures is not conditioned by the genetic code, but in the positional and metabolic relationships of these structures with the surrounding environment [10]. Several sheaths of insertion fascia are visible along the path of the vessels in the upper and lower limbs. The deep veins usually follow the path of the arteries, but the superficial veins are organized mainly in the superficial fascia. In the region of the intestine the mesentery itself is organized forming insertion inserts for the branches of the mesenteric artery and vein. However, in several places of the body these two layers merge and behave as unique, as for example in the vessels that penetrate the glands. The vascular insertion fascia is formed by organizations of mesodermal mesenchymal cells by the relationships between vessels and structures around their path [33]. The fascia of the common carotid bifurcation region have specialized structures called the carotid body. They are regions with a high concentration of free nerve endings specialized in capturing changes in pressure and oxygen concentration. This information ascends to the brain stem through the glossopharyngeal nerve [15]. Changes in the cervical fascia may interfere with this system of capturing mechanical information by these receptors [33]. The same happens in the region of the aortic arch. However, in this region, the afferent information is carried by the vagus nerve to the cardiac nucleus of the brainstem. In the fascia of the pulmonary veins, there is a large presence of free nerve endings with sensitivity to mechanical changes in the region of the pulmonary parenchyma, ascending through the X to the cranial [36].

The vessels and the lymphatic system are formed at a later stage of embryonic development. At the end of the fifth week, lymphatic vessels begin to be formed, possibly through the cells of the progenitors of venous origin due to metabolic interactions of the embryo. The formation is still not completely clear. Apparently, a centripetal formation occurs. Mesodermal cell differentiation occurs from the cardinal veins and the intersomitic veins [9]. The beginning of these formations occurs near the jugular region, forming a pair of lymph sacs. Thereafter, a similar organization occurs in the abdominal region and in other regions of the body. Cellular microvessel organizations join these structures, taking the dorsal aortic arteries as a guide. As the right dorsal aorta degenerates, possibly due to the repositioning of the transverse septum and the liver, the lymph vessels below the diaphragm follow the path of the left aorta, draining into the left subclavian vein [37].

The innervation of the vascular fascia accompanies the smooth muscle layers. The innervation of the arterial, venous and lymphatic system occurs through the sympathetic autonomic nervous system. The vascular afferents enter the same levels of origin as the sympathetic spinal cord. These afferences carry nociceptive information due to the importance of circulation to the body’s tissues [38].

Neural fasciae

**Figure 4:**
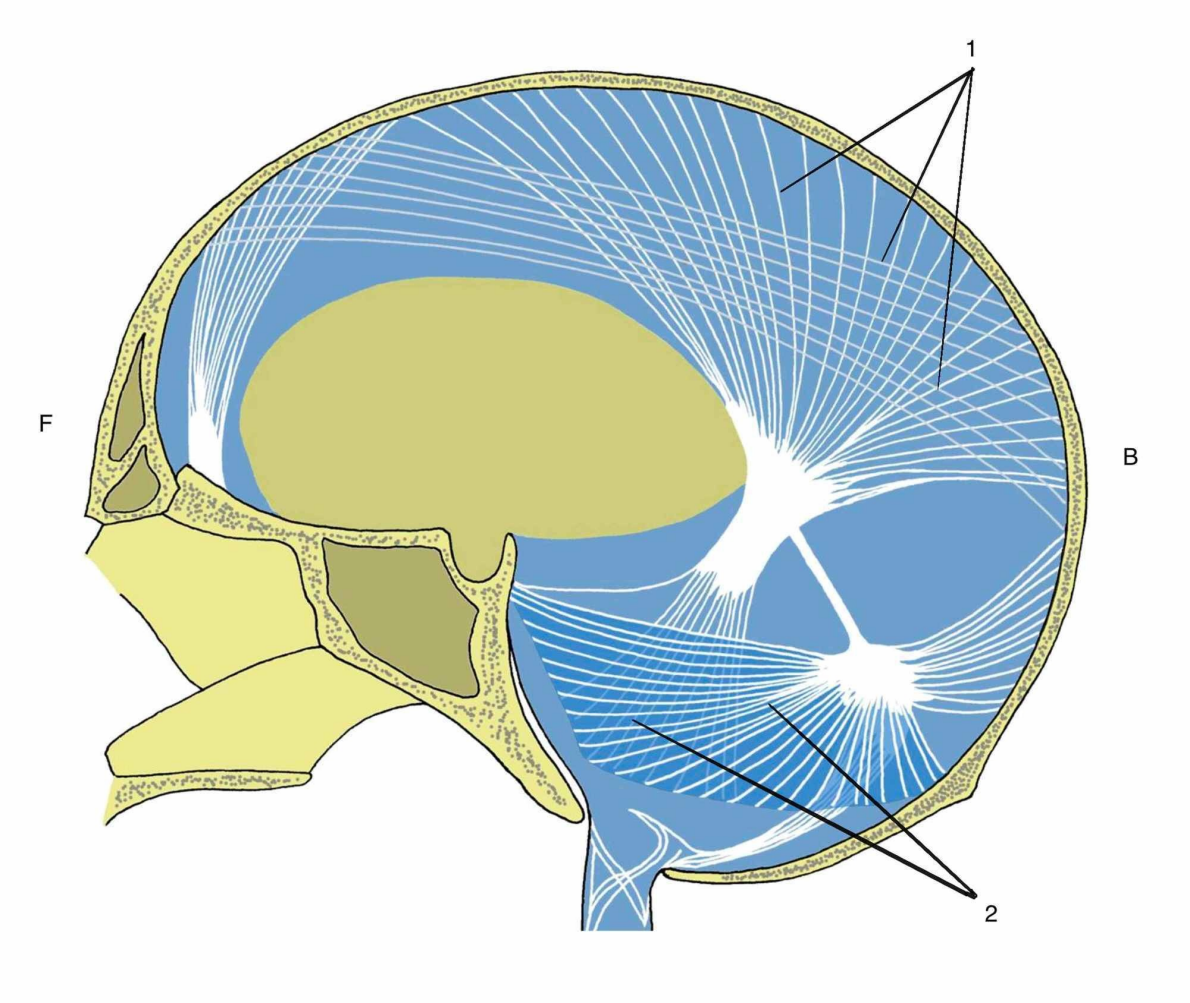
Meningeal dural bands F: front; B: back; 1: dural falx; 2: tentorium cerebelli Author's personal database image

The central nervous system develops from the neural tube [26]. At the beginning of embryonic development, the cranial part of the neural tube develops more quickly. The mesoderm close to this structure is responsible for all the metabolic support for this accelerated development. The cells juxtaposed to the neural tube organize themselves to form a primitive layer that surrounds the tube, the future dura mater or pachymeninx. From the mesoderm they also form the arteries and veins that help to provide this growth support. These structures, at the same time that they assist in the cerebralization process, cannot keep up with the growth rate of ectodermal cells, becoming a restrictive apparatus that brakes the neural tube. This difference in the speed of growth generates the first fold of the embryo, called cephalization. The fold location of the neural tube is rich in mesodermal cells forming a structure called the submesencephalic septum [10]. Above this structure the brain is being formed by the ectoderm and below the submesencephalic septum is the endoderm that will develop Foregut. In the future, the bones at the base of the skull will develop from the submesencephalic septum. All tension lines of the mesodermal layer that surround the future brain of the embryo converge to this region, being the center of several dural tension lines. These dural tension lines influence the formation of brain grooves and gyrus [10]. The dura mater is formed by the organization of mesodermal cells forming two layers of anatomically dissectible tissue [15]. A layer is intimately attached to the internal part of the skull bones, called the bone dura mater. The other layer, meningeal dura mater, is heavily influenced by the dural bands dividing the brain into anatomically well-defined compartments. The compartments are the tentorium cerebelli, falx of the brain, falx of the cerebellum and diaphragm of the sella turcica (Figure 4). The tentorium cerebelli constitutes a dural organization horizontally disposed to which it divides the cerebellum cortex. The falx of the brain is the vertical organization in the medial region of the skull, separating the brain into the right and left cerebral hemisphere [39]. The falx of the cerebellum is a vertical organization of the dura mater in the cerebellum region dividing this structure into right and left cerebellar lobes. In the region of the sphenoid body the dura mater is inserted in the clinoid processes forming the diaphragm of the local sella turcica where the pituitary gland will be located. It is a region of passage for several cerebral vessels and close to the limbic areas of the telencephalon and diencephalon [39]. The dura mater is the only meninges with nociceptive innervation. The cranial part presents a diverse innervation. Above the tentorium cerebelli the dura mater is innervated by several branches of the trigeminal nerve (V). Below the tentorium cerebelli, it is innervated by the vagus (X) nerve and by branches of the cervical plexus originating from the first two vertebral levels. The posterior part of the foramen magnum is innervated by the vagus and the anterior part by the hypoglossal nerve (XII) [15].

The innermost meninges, leptomeninges, develop from the mesenchymal ectodermal cells at the edges of the embryo’s neural tissue. The pia mater comes into direct contact with the cerebral parenchyma. It is a thin, flexible and anatomically dissectible structure. The arachnoid presents a disorganized collagenous histological pattern allowing a great flow of structures, protection and adaptation. Between the two we have the cerebrospinal fluid that provides mechanical protection and metabolic support for the brain and spinal cord [40]. This entire complex of the three meninges allows complex organization of the skull and spinal cord. At the medullary level, the meninges are organized forming several ligaments that have support functions and, at the same time, protection of the medullary nervous system. Yellow ligament, or posterior longitudinal ligament in the anterior region of the spinal canal, Trolard’s ligament in the sacroiliac region, dentate ligaments throughout the spine, cruciform and denticular ligaments in the region of the skull base, in the region of the vertebral foramen to protect and support the nerve roots [41].

In the medullary dura mater, nociceptive innervation occurs through multipolar nerves taking the information to various spinal segments, which can generate a perception of referred pain affecting even the limbs [39]. These structures connect to the mesodermal wraps that provide similar support to the peripheral nervous system, establishing a continuity relationship of the entire nervous system.

Peripheral nerves are divided into cranial, spinal and autonomic nerves. The cranial nerves, with the exception of the I and II pairs, are formed from rhombomeres, structures located in the region of the rhombencephalon at the embryonic level. Their wraps are formed by the organization of mesenchymal cells of ectodermal origin from the cranial and mesodermal neural crest [39].

The peripheral nervous system is formed from the neural crest. Neural branches to innervate blood vessels throughout the body. This innervation occurs from somite 8 to 22 forming the sympathetic autonomic nervous system. With the development of the endodermal digestive tube, blood vessels are formed from the dorsal aortae. These vessels also carry innervation of the neural crest to the visceral fasciae and the movement fasciae of the entire body. The parasympathetic nerves develop from the rhombomeres, in the case of the parasympathetic part of the vagus, oculomotor, facial and glossopharyngeal, and also through the more caudal somites, in the future sacral levels from S2 to S4 [15]. Spinal nerves are developed from neural crest cells at the medullary level. Peripheral neurons are conducted to the tissues of the body mainly by mesodermal cells that originated the smooth, striated and cardiac muscles. The mesoderm that provides metabolic support for the growth of peripheral nerves is available in such a way as to organize, protect and direct the neural pathway [10]. The connective tissue that surrounds a neural fiber is called an endoneurium, a perineurium fascicle and an epineurium nerve. Along the nerve path, in places of possible compressions, it is possible to find extra protection from loose, disorganized connective tissue, called the mesoneurium [39].

Development of neural receptors

The first neural receptors to be formed are the free nerve endings, and their axons type III and IV. These receptors are polymodal carrying information of several different orders to the central nervous system. Those who follow a destination for Somites, column tape, translate nociceptive information [42-43]. They are simple and primitive receptors, found in phylogenetically primitive animals. The most specific receptors, such as Meissner, Merkel of pressure and touch, and proprioceptives such as Ruffini, Pacini, muscle spindle and Golgi tendon organ (GTO) are encapsulated receptors, usually with fat [42]. Their information is much more specific and is carried to the spine through fast fibers, with a thick myelin sheath, type I and II. Phylogenetically they are more recent than polymodals. Embryologically they are formed in conjunction with the development of muscles, movement fasciae, dermis and superficial fascia, at a later stage in the development of polymodal receptors. Embryology repeats the pattern of phylogenesis [30].

Visceral fasciae

The endoderm forms a large part of the body's viscera, also called the primitive gut, with the exception of the genitourinary system that is formed by the intermediate mesoderm. The endodermic tube is divided into foregut, midgut and hindgut [9]. Foregut will originate the entire respiratory system, and the digestive system of the head up to the second part of the duodenum (D2). From the second part of the duodenum to the two sections of the transverse colon, it develops from the midgut. Hindgut will develop the distal-third of the transverse colon, descending colon, sigmoid, rectum and pelvic organs.

The layers of visceral smooth muscle and the two fascial layers develop from the lateral mesodeme. At first the lateral mesoderm forms a structure called the mesogastrium. The mesogastrium organizes all the growth and morphology of the visceral part of the embryo. It is divided into ventral and dorsal mesogastrium. Foregut’s fascia are derived from the ventral and dorsal mesogastrium. In midgut and hindgut, only the dorsal mesogastrium will develop in the fascial structures, as the anterior mesogastrium degenerates. This justifies the great mobility of some abdominal organs, such as the jejunum and ileum through the mesentery, which fixes only on the posterior abdomen wall [15]. As previously described, the mesoderm is the embryonic layer responsible for metabolic interactions for the development of ectodermal and endodermal structures. A part of the lateral mesoderm is related to the ectoderm, influencing and at the same time being influenced by it. This part is called somatopleure. Somatopleure will help in the formation of the dermis in conjunction with the dermatome developed through the paraxial mesoderm. The somatopleure will give rise to parietal visceral fasciae, also known as insertional fasciae [33]. All ligaments, omenta and structures that connect the visceral with the somatic system, with another visceral or with the diaphragm are products of the differentiation of this mesodermal layer. These structures have the characteristic of presenting an extracellular matrix rich in collagen fiber and with little elastin fiber, giving a stability characteristic to these relationships [36]. All of these fascia are innervated by spinal and sympathetic nerves. The thoracic part of the visceral fascia and the fascial and ligament structures just below the diaphragm receive phrenic innervation [44]. Regarding nociceptive information, they have interoceptive, polymodal receptors that also carry nociceptive information [38]. In addition, they have proprioceptors related to the perception of movement speed: the paciniform corpuscles [36]. Another part of the lateral mesoderm close to the endoderm is influenced by the metabolism of the development of these structures, presenting a different behavior from the somatopleure. This part is called splanchnopleure and will form the entire muscular part of the digestive and respiratory tract in addition to originating the innermost fascia of the viscera, also known as the investing fascia. This tissue layer provides support for the development of the digestive and respiratory tract, entering into the parenchyma, influencing the function and shape of these tissues [9,10,33]. These fascia present a matrix histological condition rich in elastin fibers in most organs with the exception of the heart and kidney, organs with relevant fluid function [36]. Its innervation basically occurs by the parasympathetic autonomic nervous system. The vagus nerve innervates the entire respiratory tube and the digestive tube up to the distal two-thirds of the transverse colon, all structures originating from foregut and midgut. The final third of the transverse colon, descending colon, sigmoid, rectum and part of pelvic organs, which develop from the hindgut will be innervated by the sacral parasympathetic levels S2-S4 [33]. Polymodal interoceptive receptors are present in this tissue helping to capture important information for the functioning of the tissues. Phylogenetically more primitive receptors without the presence of capsules and fat in their constitution. 

Regarding the receptors belonging to neurons in the sacral medulla, they carry nociceptive information. The receptors belonging to vagal afferents would not take nociceptive stimuli, which by definition would have a synapse in the posterior horn of the spinal cord [45]. The first synapse of the vagal afferent neurons occurs in the nucleus of the solitary tract in the brain stem, not participating in the direct nociceptive pathway. However, this vague information has an action on the periaqueductal gray substance that influences the nociceptive modulation of all spinal levels by a descending route.In addition, various vagal information is destined for the insular cortex, the interoceptive center of the body, usually associated with the emotional part of pain [46]. Several lines of evidence demonstrate the importance of vagal afferences in the perception of pain. Thus, despite having no relation to the direct nociceptive pathway, information from the lining fascia seems to have an intense influence on pain perception.

Much of the visceral function is performed without the direct involvement of the central nervous system. The coating fascia in conjunction with the enteric autonomic nervous system are primarily responsible for this autonomy. The nerves, ganglia and plexuses of the enteric autonomic nervous system are located in the submucosal and muscular layers of the digestive and respiratory tract [33]. They are embryologically derived from sacral parasympathetic and vagal neurons. The functioning is mainly due to mechanical changes in the parenchyma and fascial tissue of these tissues through local regulation. This generates a great advantage of saving energy and efficiency, saving brain activity in direct control of digestion [47].

Diaphragm formation

The diaphragm can be considered one of the main structures of the body. It connects to several systems. It is the primary motor of breathing, participates in gastroesophageal control, responsible for altering intracavitary pressures, draining fluids, mainly the venous and lymphatic system, interfering with visceral motricity and motility acting on the function of the organs, working as a lumbar stabilizer, important for the phonation, cough, defecation, helps with motor coordination of upper and lower limb movements, among other functions [48].

**Figure 5:**
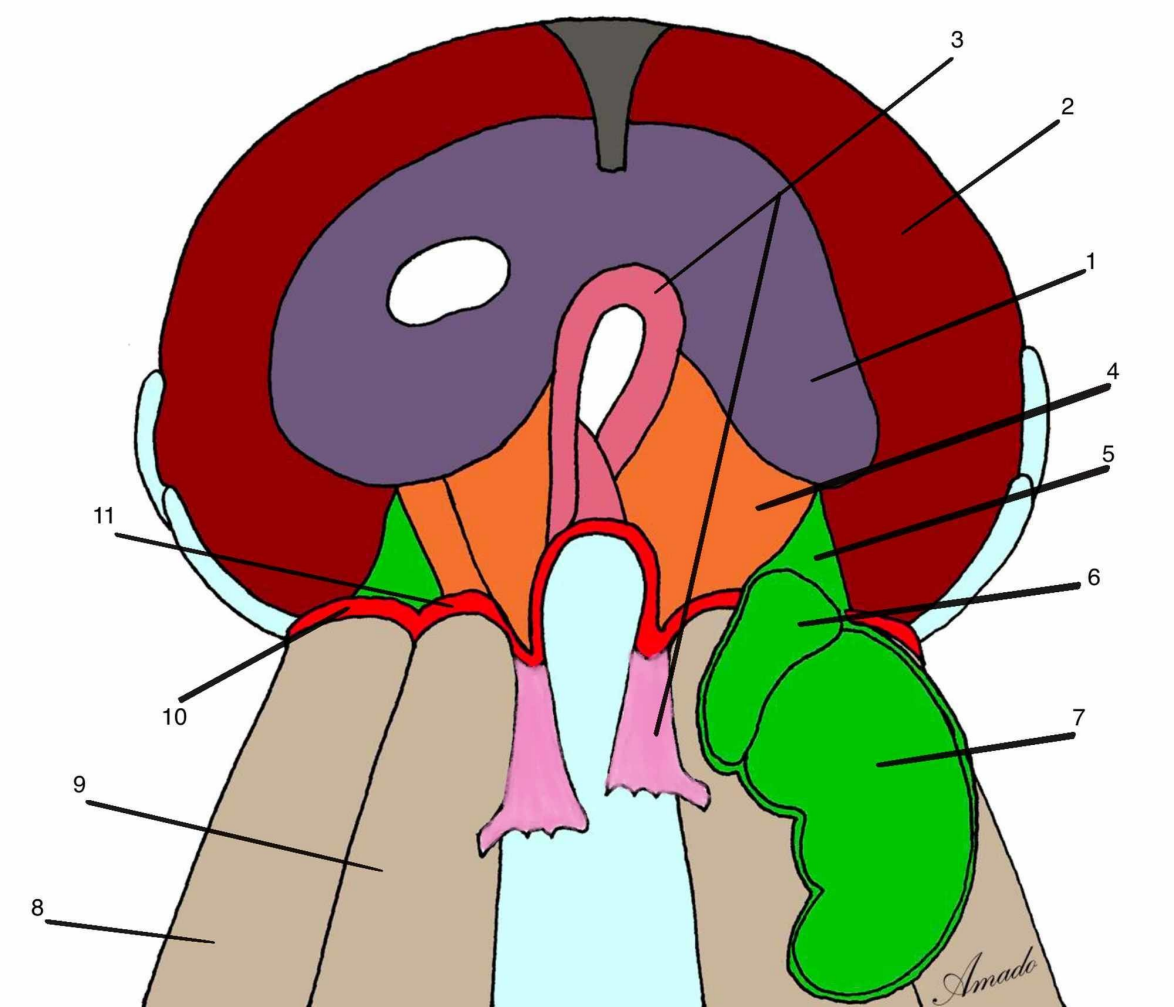
Five parts at development of diaphragm 1: phrenic center; 2: costal diaphragm; 3: crural part of diaphragm; 4: pleural mesenquimal cells; 5: suspensory ligament of adrenal gland; 6: adrenal gland; 7: kidney; 8: quadratus lumborum muscle; 9: psoas muscles; 10: lateral arcuate ligament; 11: medial arcuate ligament Author's personal database image

It is formed from five different origins, justifying its wide innervation and function in the body (Figure 5). In the first month of life, a cellular organization of mesodermal origin is formed between the primitive heart and the primitive vascular part of the liver in front of the skull. This structure is called a transverse septum. It is innervated mainly by the fourth cervical somitic level, also receiving innervation from the third and fifth level [9]. As the embryo develops, the transverse septum is repositioned together with the heart and liver, influencing the development of the thoracic, abdominal, upper and lower limbs. The transverse septum will give rise to the phrenic center or tendon center of the diaphragm. The phrenic nerve innervates the afferent and efferent part of this structure [9-15]. The posterior part of the diaphragmatic dome develops from the mesenchymal cells derived from the pleural mesoderm. The phrenic promotes the innervation of this region.

From the chest somites of the levels of T7 to T12 the costal part of the diaphragm develops. This part develops in a concentric sense, integrating with the tendon center. The costal part of the diaphragm is responsible for the bucket handle breathing movement. The afferent part of the innervation of this region occurs through the intercostal nerves from T7 to T12. The motor part comes from the phrenic [49]. The diaphragmatic crura, the pillars, and the esophageal part of the diaphragm develop from mesenchymal cells in the mesodermal part of the esophagus. This part of the diaphragm is related to several important functions such as reflux containment, lumbar stabilization, movement synchronism between the pelvic and scapular girdles. The innervation of the region close to the esophagus is vagal competence. The pillars are innervated by vagus and phrenic. An important event for the formation of the genitourinary system is the descent of the transverse septum. Thus, the development of the middle mesoderm influences the formation of a small part of the diaphragm. According to Jerome Helsmoortel, the mesodermal cells that formed the renal and suprarenal fascia help to form a posterior part of the diaphragm corresponding to the Grynfeltt triangle. The innervation of this region is phrenic [49].

Dermis and superficial fascia (pannicular fascia)

Skin is formed by the epidermis, of ectodermal origin, dermis of mesodermal origin [9]. The dermis is the structure with the highest concentration of neural receptors in the body. The dermis develops mainly from two regions of the mesoderm. From the paraxial mesoderm, from the somite we have the dermatome, which mainly develops the dermis of the posterior region of the spine. The anterior and lateral region of the trunk and the region of the dermis members develop from the lateral mesoderm, the somatopleure [15]. 

Subcutaneous tissue is the subject of debate among anatomists. Also called hypodermis, or fibrous panniculus, it consists of loose and dense connective tissue with a wide variety of adipose tissue. It usually has two distinct layers, one more fibrous and the other richer in fat [50]. The fat layers between the superficial fascia develop from mesodermal mesenchymal cells that are formed from the fifth month of gestation.

The subcutaneous tissue surrounds the whole body with the exception of the nasal, oral, orbit and anus orifices. The most superficial layer of fat, also called the superficial retinaculum of the skin, has a direct connection with the dermis. It presents collagen fibers orientation perpendicular to the skin, making this layer efficient in impact absorption. The other layer is called the deep retinaculum cutis or deep fat layer and has a direct connection with the deep fascia, a layer that involves muscle groups. This layer has collagen fibers arranged in an oblique shape, providing an important sliding function. The deep layer of fat has a dividing function between the superficial and deep layers in relation to innervation [23]. The fat layers between the superficial fascia develop from mesodermal mesenchymal cells that are formed from the fifth month of pregnancy. These layers have different characteristics depending on the region of the body and are usually divided by a fibrous lamina, the “superficial fascia” itself, which constitutes a mesenchymal cellular organization of somitic origin. Histological analyzes of the superficial fascia show a large amount of collagen fibers and elastin in its constitution [50]. The superficial fascia and the retinaculum of the skin form a three-dimensional network between the fat lobules of the hypodermis, like a hive [48]. This arrangement allows for independence and at the same time connection between the deep layers of movement and the skin. It contains a rich innervation and a large number of vessels, mainly the lymphatic and venous system. About 70% of the lymphatic vessels and superficial veins are in this layer [50]. In regions such as the hands, feet, sternum, and face, the superficial fascia is unified with the deep fascia, making it impossible to separate into isolated layers [17]. In non-primate mammals, the presence of muscles occurs in this subcutaneous layer. These muscles have a function more associated with skin adaptations than properly linked to joint movements. In primates, especially in Homo sapiens, we only find muscles in the subcutaneous tissue in the neck and face [27]. In the anterior region of the neck we have the platysma muscle in the superficial fascia, which has an embryonic origin, usually at weeks nine to 10 from the cervical lamina and the mandibular extension from the first pharyngeal arch. It plays an important role in the venous and lymphatic drainage of the neck region. It presents continuity with the risorius muscle, of the mimic musculature [17]. The muscles of the mimic musculature are found in this subcutaneous tissue and have an important function related to the ability to socialize.

The muscles of the face and superficial fascia are mainly derived from the mesenchymal cells of the neural crest originating from the second pharyngeal arch, around the sixth to seventh week of development [11].

The interaction between the dermis and the subcutaneous tissue is one of the most important factors for the success of Homo sapiens. The wide variety of neural receptors promotes a perception of the external environment. In addition, we have about 5 to 10 million sweat glands in the dermis, spread throughout the body, unlike other primates. When sweat comes into contact with air it cools the skin and the blood that passes through the superficial fascia. This blood, as it travels through the body, cools the organs and the brain, constituting an efficient mechanism for controlling temperature on exertion, which is extremely important for survival. The sweat and sebaceous glands develop from cells of ectodermal origin in the skin, migrating to the dermal region [27].

## Conclusions

The fascial system is formed mainly by mesodermal cells originating a wide variety of tissues in the body. The exception in mammals corresponds to the formation of the fasciae of the face and the anterior region of the neck that is formed from the mesenchymal cells originating from the ectodermal cranial neural crest. The development of the neural system is a major influencer and at the same time it is influenced by this great fascial system, establishing an intimate relationship, called the neurofascial system. Despite the division of the topics present in this text, the fascial system is indivisible. The tissues formed do not follow the didactic divisions proposed by linear models. They participate in the formation of tissues formed from the endoderm and ectoderm, relating directly to the parenchyma of the structures. At a microscopic level, the entire body is interconnected by this large fascial network, making it a fast and extremely efficient communication system for life.

A lot of conclusive research is still missing regarding the formation and interaction of this great fascial system. We hope that this text will help future works in order to clarify the formation of the system that represents this link of the whole body.
